# Chase-away evolution maintains imperfect mimicry in a brood parasite–host system despite rapid evolution of mimics

**DOI:** 10.1038/s41559-023-02232-4

**Published:** 2023-10-23

**Authors:** Tanmay Dixit, Jess Lund, Anthony J. C. Fulford, Andrei L. Apostol, Kuan-Chi Chen, Wenfei Tong, William E. Feeney, Lazaro Hamusikili, John F. R. Colebrook-Robjent, Christopher P. Town, Claire N. Spottiswoode

**Affiliations:** 1 Department of Zoology, University of Cambridge, Cambridge, UK; 2 DST-NRF Centre of Excellence at the FitzPatrick Institute of African Ornithology, University of Cape Town, Rondebosch, South Africa; 3 Computer Laboratory, University of Cambridge, Cambridge, UK; 4 Behavioural and Evolutionary Ecology Group, Doñana Biological Station (CSIC), Seville, Spain; 5 Department of Biosciences, Durham University, Durham, UK; 6 Musumanene Farm, Choma, Zambia

**Keywords:** Behavioural ecology, Community ecology

## Abstract

We studied a brood parasite–host system (the cuckoo finch *Anomalospiza imberbis* and its host, the tawny-flanked prinia *Prinia subflava*) to test (1) the fundamental hypothesis that deceptive mimics evolve to resemble models, selecting in turn for models to evolve away from mimics (‘chase-away evolution’) and (2) whether such reciprocal evolution maintains imperfect mimicry over time. Over only 50 years, parasites evolved towards hosts and hosts evolved away from parasites, resulting in no detectible increase in mimetic fidelity. Our results reflect rapid adaptive evolution in wild populations of models and mimics and show that chase-away evolution in models can counteract even rapid evolution of mimics, resulting in the persistence of imperfect mimicry.

## Abstract (web summary)

Using a 50 year time series of photos of cuckoo finch eggs and those of its host, prinia, the authors document that cuckoo eggs evolve towards prinia eggs, but progressive evolution of prinia eggs away from cuckoo eggs results in no detectible increase in mimetic fidelity.

## Main

Ever since Bates observed the remarkable similarity between different South American butterfly species^1^, the phenomenon of mimicry has been used to illustrate how natural selection can produce striking adaptations. For mimicry to exist, mimics must evolve to resemble models. If models benefit from being discriminable from mimics, ‘chase-away’ selection should drive models to evolve away from mimics^2^. Thus, chase-away evolution could prevent the accuracy of mimetic resemblance, termed mimetic fidelity, from increasing over time^3,4^. However, few studies have examined evolutionary trajectories of both models and mimics simultaneously, probably because the required long-term data are difficult to obtain. To our knowledge, the only study to have examined changes in mimetic fidelity over time found that, for one of four traits studied, mimetic fidelity increased over time^5^. While this might suggest that chase-away selection was insufficient to prevent increases in mimetic fidelity over time, it is unknown whether the trait is used in discriminating between models and mimics and thus whether observed patterns were due to selection in the context of mimicry. Here, we study an aggressive mimicry system over 50 years to test the hypothesis that chase-away selection on models prevents increases in mimetic fidelity over time.

**Fig. 1:**
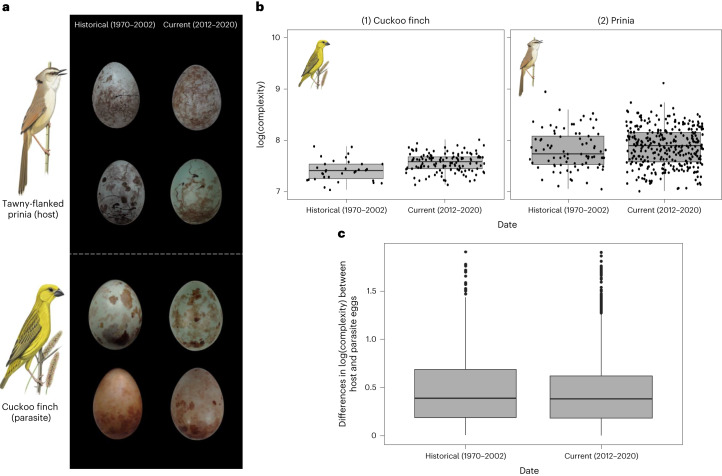
Changes in egg pattern complexity over time. **a**, Randomly selected host (above) and parasitic (below) eggs, from the historical (left) and current (right) samples. **b**, Changes in egg pattern complexity (log-transformed) over time in (1) parasites (*n* = 162 biologically independent eggs) and (2) hosts (*n* = 414 biologically independent eggs). Boxes range from the 25th to the 75th percentile and horizontal lines represent medians. Minima and maxima are defined by the smallest datapoint no lower than the 25th percentile minus 1.5× interquartile range (IQR) and the largest datapoint no greater than the 75th percentile plus 1.5× IQR, respectively. All datapoints are shown as dots. **c**, Mimetic fidelity through time: no significant change in differences in log(complexity) between all historical (*n* = 2,788) and current (*n* = 42,496) pairs of parasites and hosts These pairs were generated from the 162 parasitic eggs and 414 host eggs photographed. Boxes range from the 25th to the 75th percentile and horizontal lines represent medians. Minima and maxima are defined by the smallest datapoint no lower than the 25th percentile minus 1.5× IQR and the largest datapoint no greater than the 75th percentile plus 1.5× IQR, respectively. Outliers are shown as dots. Individual points are excluded as they obscure the boxplot. Bird illustrations reproduced with permission from faansiepeacock.com.

The cuckoo finch *Anomalospiza imberbis* lays eggs which imperfectly mimic the complex and variable patterns of eggs of its host, the tawny-flanked prinia *Prinia subflava* (Methods), which reject mismatched eggs from their nests^6^. Individual prinias lay eggs with distinct colour and pattern phenotypes (egg signatures; Fig. 1a), such that a given cuckoo finch egg will be a poor match to most prinia clutches in the population^6^. Cuckoo finch eggs (mimics) exhibit simpler patterns than prinia eggs (models) and differences in pattern complexity predict egg rejection by prinias^7^. Egg rejection therefore has fitness consequences for both hosts and parasites and this implies that selection should favour parasites evolving towards hosts (that is, evolving increased complexity) and hosts evolving away from parasites (that is, also evolving increased complexity). By quantifying pattern complexity of 414 prinia and 162 cuckoo finch eggs from 1970 to 2020 (Methods), we tested whether host and parasitic phenotypes have changed in the predicted direction in the recent past and whether such reciprocal evolution led to any change in mimetic fidelity over time. We measured complexity (a synthetic measure of several pattern traits; Methods) on a logarithmic scale, since hosts perceive this measure of complexity according to Weber’s Law^7^. Because effect sizes on logarithmic scales are not intuitive, we provide estimates as percentages where appropriate.

**Extended Data Fig. 1:**
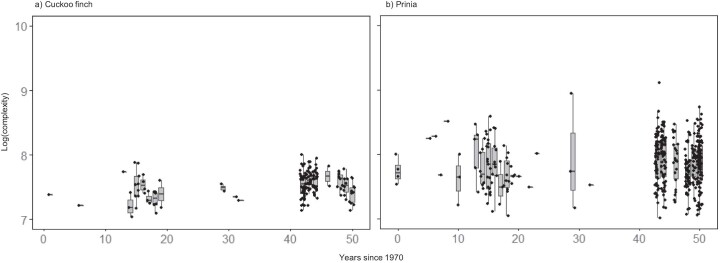
Changes in complexity across 50 years in cuckoo finches and prinias. Change in complexity over time in **(a)** cuckoo finches and **(b)** prinias. Years are defined as number of years after 1970 (such that Year 0 corresponds to 1970 and Year 50 to 2020).

A linear model confirmed that in this dataset, prinia eggs are more complex than cuckoo finch eggs (estimate = 58%, 95% confidence interval (CI) = 30–93%, *t*_572_ = 4.3, *P* < 0.001). Complexity across both species increased slightly but significantly over 50 years (estimated increase = 0.5%, 95% CI = 0.05–0.9%, *t*_572_ = 2.0, *P* = 0.04; Fig. 1b and Extended Data Fig. 1). There was no significant difference between species in the rate of increase in complexity (interaction between species and year during which the egg was laid: estimate = −0.4%, 95% CI = −0.9–0.1%, *t*_572_ = −1.4, *P* = 0.15). Because heteroscedasticity in the data (that is, hosts exhibiting higher variance in complexity than parasites, probably as a result of diversifying selection on host phenotypes^6,8^) may invalidate model inferences, we bootstrapped the linear model (Methods). As a further validation, we categorized eggs into those laid from 1970 to 2002 (historical; predominantly 1980–1990) and those from 2012 to 2020 (current). All results were consistent with the original model (Methods). Overall, the finding that complexity increases over time suggests that parasites have evolved towards hosts and that hosts have evolved away from parasites at a similar rate.

If chase-away evolution in hosts occurred at a similar rate to parasite evolution, as implied above, then we would expect to see limited increases in mimetic fidelity despite rapid evolution of parasites. To quantify changes in mimetic fidelity, we calculated all host–parasite complexity differences from 1970 to 2002 (historical) and from 2012 to 2020 (current) (Methods). Bootstrapped estimates of historical and current mimetic fidelity showed considerable overlap (mean historical complexity difference on a logarithmic scale = 0.46, 95% CI = 0.38–0.55; mean current complexity difference = 0.42, 95% CI = 0.39–0.45; Fig. 1c). This corresponds to no significant increase in this trait-based measure of mimetic fidelity (bootstrapped estimated increase = 4%, 95% CI = −3–12%; Fig. 1c). We also independently estimated mimetic fidelity using a discriminant analysis based on complexity. The discriminant analysis for historical eggs correctly assigned 72% of eggs to the correct species (bootstrapped 95% CI = 63–80%). There was no significant difference between this and the performance of the discriminant analysis for current eggs (mean increase in mimetic fidelity = 2%; bootstrapped 95% CI = −10–13%), which assigned 70% of eggs to the correct species (bootstrapped 95% CI = 61–78%). This echoes the result of comparing pairwise combinations of eggs: both measures of mimetic fidelity indicate that no observable increase in mimetic fidelity occurred, as expected given the lack of any significant difference between hosts and parasites in the rate of change of pattern complexity over time. Thus, chase-away selection driving host evolution away from parasites probably explains why mimicry of pattern complexity remains imperfect in this host–parasite system.

Although observed changes in complexity conformed to a priori predictions of coevolution, this study is correlational. We must therefore consider alternative explanations which could influence host and parasitic eggs in tandem, such as selection on egg pattern complexity from predation or climate. However, the main predators at our field site are snakes, which rely mostly on olfaction and infrared, and prinia nests are enclosed, limiting egg visibility at long range^8^. Climate change also appears unlikely to select for increases in complexity, since increased temperatures are likely to select for fewer pattern markings (which absorb more heat than unmarked eggshells)^9^. Complexity is highly correlated with the number of pattern markings and weakly correlated with pattern coverage^7^; thus, increased ambient temperatures due to climate change should select for reduced complexity, contrary to our findings.

In summary, tracking model and mimic phenotypes over 50 years showed that despite rapid evolution of parasites, there was no detectible increase in their mimetic fidelity to hosts. This suggests that the coevolutionary response in hosts was strong enough to prevent increases in mimetic fidelity and so supports the hypothesis that the persistence of imperfect mimicry can be explained by chase-away evolution in models^3,4^.

## Methods

### Study species

At our study sites, on Semahwa and Musumanene Farms (around 16.74º S, 26.90º E) and surrounding areas in the Choma District of southern Zambia, the cuckoo finch currently parasitizes four cisticolid warbler species^10^. Of these four species, tawny-flanked prinias are the commonest, and have the most variable (and subjectively the most complex) egg patterns^11^. High interindividual variation in prinias (i.e. the presence of egg signatures) provides an effective defence against parasites, since egg signatures facilitate the rejection of mismatched eggs from host nests^6,12^. Although many egg signature traits may be important for egg rejection in this system^6^, we focussed on complexity because quantifiable differences between hosts and parasites in this trait allow us to make clear predictions about the direction of evolution, namely that both should evolve towards higher complexity^7^.

### Photography of eggs

In all analyses, one egg per photographed clutch was included (prinia *n* = 414; cuckoo finch *n* = 162; from 1970 to 2020), with a single image considered representative of the egg’s phenotype. Images of eggs collected from 1970 to 2002 (from the private collection of J.F.R.C.-R., collected by J.F.R.C.-R. and L.H. and deposited in the Livingstone Museum, Zambia) were taken by C.N.S. Most of these eggs were from the 1980s. Images from 2013 were taken by W.E.F., C.N.S. and W.T.; images from 2014 were taken by W.T. and C.N.S.; images from 2018 to 2020 were taken by T.D.; all other images were taken by C.N.S. Although host and parasite eggs were also studied in 2007–2009^6^, these years were excluded from this study because images from 2007 to 2009 were not comparable to other images taken (due to differences in scaling and normalization^7^). In a few years, some host eggs were not photographed or analysed due to a specific research focus on parasitic eggs, and host eggs were not routinely photographed owing to time constraints. Parasitic eggs can be reliably distinguished from host eggs by the absence of ‘scribbles’ of pigment on their shells, which hosts always exhibit^13^. Images were taken in linearized RAW format, in shade with either a Nikon D90 camera with a 60 mm Micro-Nikkor lens or a Fuji Finepix S7000 camera. For eggs collected from 1970 to 2002, a 17% grey card was used to normalize images. For all other eggs, two grey standard squares (N6.5 and N5; reflectance values 36.2% and 19.8%, respectively) of an X-rite ColorChecker Passport (X-Rite) were used to normalize images.

In all images except for those from 2018 to 2020, only ‘one side’ of each egg was photographed. In 2018–2020, eggs were photographed four times, rotating the egg through 90° around the long axis after each image, to maximize the amount of pattern photographed^7,14^. This produced images of ‘sides’ a, b, c and d, where a is opposite c and b opposite d. When determining historical changes, we used complexity values from only one side of eggs photographed in 2018–2020 (side a), rather than the average of a and c as used previously^7^. Complexity values for different sides of the egg are highly repeatable^7^.

### Image analysis

We used the MICA toolbox^15^ in ImageJ to normalize and scale images to 29 px mm^−1^, ‘cut out’ (i.e. remove from the background) and mask (i.e. add an artificial black background to) eggs and produce greyscale images from the green channel. The green channel was used because it corresponds closely to the sensitivity of avian double cones, thought to be involved in pattern processing^16^. Pattern features were extracted using NATUREPATTERNMATCH (NPM)^17^. NPM detects and encodes local features (SIFT features) as 132-dimensional vectors, which loosely correspond to pattern markings. Complexity of the egg pattern was then calculated as in ref. ^7^. Briefly, six traits were measured: (1) the number of pattern features, (2) the variation in position of features on eggs, (3) the variation in the scale (size) of features, (4) the variation in the orientation of features, (5) the Redies change, a measure of how much intensity (brightness) changes across an image and (6) a measure of clustering tendency of features and within-cluster feature variation. All but trait (5) were based on features extracted using NPM. An optimization algorithm optimized the complexity metric (defined as a linear combination of these six traits) such that the absolute complexity difference between an experimental egg and the host clutch in which it was placed would best predict rejection of the experimental egg. For full details of this quantification, see ref. ^7^. Although this metric was based on present-day rejection data, we found evidence that selection has acted on host and parasite pattern complexity in the recent past (see main text). This implies that the complexity metric is not only relevant to current host rejection behaviour but also was relevant to rejection behaviour in the recent past.

Because perception conforms to Weber’s Law^7^ (that is, hosts perceive relative, rather than absolute, differences in complexity), we quantified pattern complexity on a logarithmic scale, with estimates of percentage changes in complexity calculated as exp(estimate).

One concern with using historical egg collections is that the background colour of eggs can fade over time, especially if they are poorly stored, which was not the case for the eggs photographed as part of this study. Old eggs were photographed in 2007 and 2009 and eggs were kept in a darkened room and collected relatively recently^8^. Furthermore, it is largely blue-green colours on eggs which fade (for example, ref. ^18^), which has no relevance to the pattern measures we extracted, since pattern measures extracted from NPM should be unaffected by the underlying colour. In the unlikely event that fading affected the detectability of faint markings by NPM, any background colour fading on old eggs would make faint markings more detectable on these eggs, resulting in higher complexity scores for old eggs than for fresh eggs. Our results run counter to this (see main text) and are therefore conservative.

A second concern with studying host and parasitic egg phenotypes more generally is that some (probably poorly matched) parasitic eggs may be rejected from host nests before data from that nest are collected. This may mean that only closely matched parasitic eggs are phenotyped. However, in this system this is unlikely to be a problem, since (1) hosts often take 1–4 days to reject a poorly matched egg (particularly eggs that are poorly matched in terms of pattern, rather than colour)^7^ and (2) high variation in host egg appearance between clutches (Fig. 1b) means that all cuckoo finch eggs are poor matches to most of the host population at any given time. Thus, there is unlikely to be a bias towards phenotyping well-matched eggs.

### Testing for changes in complexity over time

All statistical analyses were conducted in R (v.4.0.2; ref. ^19^). We used linear models (function lm) to quantify change in complexity over time across species, using the model complexity ~ species + year + species : year. For example, a negative coefficient for the interaction term, with positive coefficients for the species and year terms would indicate that prinia complexity was greater than cuckoo finch complexity and that complexity increased over time but cuckoo finch complexity increased more than prinia complexity.

First, we tested for continuous changes over time. Year was modelled as a continuous variable with years assigned integer values from 0 (year 1970) to 50 (year 2020). Because prinias exhibited much greater variance than cuckoo finches (thus falsifying the model assumption of homoscedasticity), we bootstrapped the model to calculate 95% CI for model coefficients using 1,000 replicates. Results from bootstrapping were consistent with the initial model. CIs for the interaction term spanned zero (estimate = −0.4%, 95% CI = −0.7–0.003%), while CI for species (estimate = 58%, 95% CI = 35–86%) and year (estimate = 0.5%, 95% CI = 0.2–0.7%) did not span zero, indicating that prinia eggs are more complex than cuckoo finch eggs and that complexity increased over time. Median complexity appeared to fluctuate during certain periods (Extended Data Fig. 1), such that the overall increase in complexity was not monotonic. These fluctuations are probably due to low sample sizes of eggs from specific years, combined with very high population-wide variation in complexity. Such sampling error is especially likely during periods such as the mid- to late-1980s, in which sample sizes were low for each year; correspondingly, fluctuations in complexity were apparent in these years. However, with these data we cannot rule out other selective pressures or environmental influences driving short-term increases or decreases in complexity in one or both species. Regardless of the cause of these apparent fluctuations, they mean that we did not observe a monotonic increase in complexity in either species. Therefore, we conducted further analyses to test the robustness of the results of the linear model.

We subdivided the datasets into historical eggs (from 1970 to 2002; prinia *n* = 82, cuckoo finch *n* = 34) and current eggs (from 2012 to 2020; prinia *n* = 332, cuckoo finch *n* = 128). Results for this model were also consistent with the previous models (species—estimate = 34%, 95% CI = 23–45%, *t*_572_ = 8.4, *P* < 0.001; year—estimate = 16%, 95% CI = 2–32%, *t*_572_ = 2.3, *P* = 0.02; interaction—estimate = −9%, 95% CI = −22–7%, *t*_572_ = −1.3, *P* = 0.2). Conclusions also remained unchanged when this model was bootstrapped (Species—estimate = 34%, 95% CI = 27–42%; year—estimate = 16%, 95% CI = 7–26%; interaction—estimate = −9%, 95% CI = −19–3%).

In summary, complexity was higher in prinias than cuckoo finches, complexity increased over time across both species and there was no detectible difference between species in the rate of increase of complexity over time.

### Testing for changes in mimetic fidelity over time

We measured mimetic fidelity using two methods. The first method calculated all host–parasite differences in each time period. Comparing all host–parasite pairs assumes that cuckoo finches lay their eggs at random in prinia nests; that is, independently of the patterning on the prinia eggs they contain. This has been shown to be a valid assumption in this system^6^. All possible pairings of parasite and host eggs measured at each time point (*n* = 82 × 34 = 2,788 for historical data; *n* = 332 × 128 = 42,496 for current data) provide a sample from the joint distribution of parasite and host pairs in the population at large during each time period. To test whether mimetic fidelity had changed over time, we generated absolute differences in the logarithm of complexity between all pairs of host and parasitic eggs. From these we calculated the mean absolute difference for each time period and used a two-sample bootstrap with 500 replicates to estimate its 95% CI (mean ± 2 × bootstrap s.e.m.). The bootstrap was necessary because the number of paired differences contributing to the mean estimate far exceeded the available degrees of freedom: for instance, the sample size for current data (*n* = 42,496) was generated from 460 observations (d.f. = 459). Thus, simply conducting statistical tests without a bootstrap would overestimate statistical power, whereas bootstrapping allows calculation of confidence intervals which do not overestimate statistical power.

As a second measure of mimetic fidelity, we used flexible discriminant analysis (FDA; function fda in the R package mda^20^) with log(complexity) as the only predictor and with uninformed (that is, equal and unbiased) priors. A high-performing FDA would indicate low mimetic fidelity because high performance would imply that the algorithm can accurately assign eggs to species. Since the performance of an FDA tends to increase with sample size, we resampled current eggs to the same number as historical eggs (*n* = 82 prinia and *n* = 34 cuckoo finch). We ran 1,000 iterations of the FDA for both historical and current populations to calculate confidence intervals.

The two measures of mimetic fidelity used here correspond to slightly different questions. The mean host–parasite pairwise distance is an estimate of the average similarity (in terms of complexity) of a randomly selected host–parasite pair, for the historical and present-day subsets. This simulates the visual information available to guide the behaviour of a host female, who must compare her own egg(s) with the egg of a parasite. The FDA provides an estimate of the likelihood of assigning eggs correctly to species based on their complexity, for each subset. This considers whether mimetic fidelity has changed at a population level.

### Reporting summary

Further information on research design is available in the Nature Portfolio Reporting Summary linked to this article.

### Supplementary information

Reporting Summary

Peer Review File

## Data Availability

All data are available at 10.17863/CAM.101483 (ref. ^21^).

## Code availability

All code is available at 10.17863/CAM.101483 (ref. ^21^).
