# Supplementary material for: Chase-away evolution maintains imperfect mimicry in a brood parasite–host system despite rapid evolution of mimics
Source: Nat Ecol Evol. 2023 Oct 23;7(12):1978–82. doi: 10.1038/s41559-023-02232-4 (PMC10697838; doi:10.1038/s41559-023-02232-4)
Supplement: Supplementary file 1 — Reporting Summary [file 41559_2023_2232_MOESM1_ESM.pdf]

nature portfolio

Corresponding author(s): Tanmay Dixit

Last updated by author(s): Sep 14, 2023

Reporting Summary

Nature Portfolio wishes to improve the reproducibility of the work that we publish. This form provides structure for consistency and transparency in reporting. For further information on Nature Portfolio policies, see our Editorial Policies and the Editorial Policy Checklist.

Statistics

For all statistical analyses, confirm that the following items are present in the figure legend, table legend, main text, or Methods section.

n/a	Confirmed	
☐	☒	The exact sample size (*n*) for each experimental group/condition, given as a discrete number and unit of measurement
☐	☒	A statement on whether measurements were taken from distinct samples or whether the same sample was measured repeatedly
☐	☒	The statistical test(s) used AND whether they are one- or two-sided<br>*Only common tests should be described solely by name; describe more complex techniques in the Methods section.*
☐	☒	A description of all covariates tested
☐	☒	A description of any assumptions or corrections, such as tests of normality and adjustment for multiple comparisons
☐	☒	A full description of the statistical parameters including central tendency (e.g. means) or other basic estimates (e.g. regression coefficient) AND variation (e.g. standard deviation) or associated estimates of uncertainty (e.g. confidence intervals)
☐	☒	For null hypothesis testing, the test statistic (e.g. *F*, *t*, *r*) with confidence intervals, effect sizes, degrees of freedom and *P* value noted<br>*Give P values as exact values whenever suitable.*
☒	☐	For Bayesian analysis, information on the choice of priors and Markov chain Monte Carlo settings
☒	☐	For hierarchical and complex designs, identification of the appropriate level for tests and full reporting of outcomes
☐	☒	Estimates of effect sizes (e.g. Cohen's *d*, Pearson's *r*), indicating how they were calculated

Our web collection on statistics for biologists contains articles on many of the points above.

Software and code

Policy information about availability of computer code

Data collection	No software was used to collect data in this study.
Data analysis	All analyses were conducted in R version 4.0.2

For manuscripts utilizing custom algorithms or software that are central to the research but not yet described in published literature, software must be made available to editors and reviewers. We strongly encourage code deposition in a community repository (e.g. GitHub). See the Nature Portfolio guidelines for submitting code & software for further information.

Data

Policy information about availability of data

All manuscripts must include a data availability statement. This statement should provide the following information, where applicable:

- Accession codes, unique identifiers, or web links for publicly available datasets
- A description of any restrictions on data availability
- For clinical datasets or third party data, please ensure that the statement adheres to our policy

All data are available at https://doi.org/10.17863/CAM.101483.

Research involving human participants, their data, or biological material

Policy information about studies with human participants or human data. See also policy information about sex, gender (identity/presentation), and sexual orientation and race, ethnicity and racism.

Reporting on sex and gender	*Use the terms sex (biological attribute) and gender (shaped by social and cultural circumstances) carefully in order to avoid confusing both terms. Indicate if findings apply to only one sex or gender; describe whether sex and gender were considered in study design; whether sex and/or gender was determined based on self-reporting or assigned and methods used. Provide in the source data disaggregated sex and gender data, where this information has been collected, and if consent has been obtained for sharing of individual-level data; provide overall numbers in this Reporting Summary. Please state if this information has not been collected. Report sex- and gender-based analyses where performed, justify reasons for lack of sex- and gender-based analysis.*
Reporting on race, ethnicity, or other socially relevant groupings	*Please specify the socially constructed or socially relevant categorization variable(s) used in your manuscript and explain why they were used. Please note that such variables should not be used as proxies for other socially constructed/relevant variables (for example, race or ethnicity should not be used as a proxy for socioeconomic status). Provide clear definitions of the relevant terms used, how they were provided (by the participants/respondents, the researchers, or third parties), and the method(s) used to classify people into the different categories (e.g. self-report, census or administrative data, social media data, etc.) Please provide details about how you controlled for confounding variables in your analyses.*
Population characteristics	*Describe the covariate-relevant population characteristics of the human research participants (e.g. age, genotypic information, past and current diagnosis and treatment categories). If you filled out the behavioural & social sciences study design questions and have nothing to add here, write "See above."*
Recruitment	*Describe how participants were recruited. Outline any potential self-selection bias or other biases that may be present and how these are likely to impact results.*
Ethics oversight	*Identify the organization(s) that approved the study protocol.*

Note that full information on the approval of the study protocol must also be provided in the manuscript.

Field-specific reporting

Please select the one below that is the best fit for your research. If you are not sure, read the appropriate sections before making your selection.

☐ Life sciences ☐ Behavioural & social sciences ☒ Ecological, evolutionary & environmental sciences

For a reference copy of the document with all sections, see nature.com/documents/nr-reporting-summary-flat.pdf

Ecological, evolutionary & environmental sciences study design

All studies must disclose on these points even when the disclosure is negative.

Study description	This study measured the pattern complexity of 414 tawny-flanked prinia and 162 cuckoo finch eggs from 1970–2020, testing whether complexity differed between the species and whether and how complexity had changed over time.
Research sample	The research sample was images taken of 414 tawny-flanked prinia and 162 cuckoo finch eggs from 1970–2020, all taken on Semahwa and Musumanene Farms (around 16.74'S, 26.90'E) and surrounding areas in the Choma District of southern Zambia. These were all images available of eggs of these species, and all images were taken largely to test other hypotheses. Images of eggs collected from 1970–2002 (from the private collection of JFRCR, collected by JFRCR and LH, and deposited in the Livingstone Museum, Zambia) were taken by CNS. Most of these eggs were from the 1980s. Images from 2013 were taken by WEF, CNS, and WT; images from 2014 were taken by WT and CNS; images from 2018–2020 were taken by TD; all other images were taken by CNS. [All abbreviations refer to authors in the author list.]
Sampling strategy	All available images were used. We used bootstrapping to confirm that results were not unduly influenced by sample size.
Data collection	Images were taken in linearised RAW format, in shade with either a Nikon D90 camera with a 60 mm Micro-Nikkor lens or a Fuji Finepix S7000 camera. For eggs collected from 1970–2002, a 17% grey card was used to normalise images. For all other eggs, two grey standard squares (N6.5 and N5; reflectance values 36.2% and 19.8% respectively) of an X-rite ColorChecker Passport (X-Rite, MI, USA) were used to normalise images. Nests were located by a team of nest-finders; one egg per clutch was used in any analysis to avoid pseudoreplication.
Timing and spatial scale	Data were collected from 1970–2020, with periods of peak collection in the 1980s and 2010s, and several years with no collection, particularly years during the 1990s and 2000s. Data were not collected with this study in mind, and therefore we could only rely on data that had been collected for other purposes, hence the uneven temporal distribution of data. Data were collected from an area of approximately 3000 hectares, including on Semahwa and Musumanene Farms (around 16.74'S, 26.90'E) and surrounding areas in the Choma District of southern Zambia.
Data exclusions	One egg per clutch was randomly selected to be used in analyses, in order to avoid pseudoreplication.

Reproducibility	This study did not involve experimental findings. We conducted bootstrapped analyses, which allowed us to calculate 95% confidence intervals.
Randomization	Randomization is not relevant to this study as it was not an experimental study and there was no assignment to groups.
Blinding	All data were collected without knowledge of the hypotheses (i.e., all egg images were taken for other purposes, and there was no bias towards photographing eggs of any particular pattern complexity). The hypotheses were generated prior to data analysis.

Did the study involve field work? ☒ Yes ☐ No

Field work, collection and transport

Field conditions	Fieldwork was conducted on farmland and adjacent land in Zambia.
Location	Data were collected from an area of approximately 3000 hectares, including on Semahwa and Musumanene Farms (around 16.74'S, 26.90'E) and surrounding areas in the Choma District of southern Zambia.
Access & import/export	Analyses involved photographed eggs either from a historical egg collection (now housed in the Livingstone Museum, Zambia) or from fieldwork in which eggs were returned to nests after photography. No samples were exported. Fieldwork was conducted under permits from the Zambian Department of National Parks and Wildlife (DNPW; previously Zambian Wildlife Authority) under permit number DNPW/8/27/1.
Disturbance	No disturbance was caused when photographing eggs from the historical egg collection, as these had already been removed from natural settings. When eggs were - more recently - photographed in the field, this process was conducted quickly (<10 minutes for a clutch) to minimise any disturbance to the nesting birds. Neither species studied are of conservation concern.

Reporting for specific materials, systems and methods

We require information from authors about some types of materials, experimental systems and methods used in many studies. Here, indicate whether each material, system or method listed is relevant to your study. If you are not sure if a list item applies to your research, read the appropriate section before selecting a response.

Materials & experimental systems

n/a	Involved in the study	
☒	☐	Antibodies
☒	☐	Eukaryotic cell lines
☒	☐	Palaeontology and archaeology
☒	☐	Animals and other organisms
☒	☐	Clinical data
☒	☐	Dual use research of concern
☒	☐	Plants

Methods

n/a	Involved in the study	
☒	☐	ChIP-seq
☒	☐	Flow cytometry
☒	☐	MRI-based neuroimaging
